# Ocular following responses of the marmoset monkey are dependent on postsaccadic delay, spatiotemporal frequency, and saccade direction

**DOI:** 10.1152/jn.00126.2023

**Published:** 2023-06-28

**Authors:** Hoi Ming Ken Yip, Timothy John Allison-Walker, Shaun Liam Cloherty, Maureen Ann Hagan, Nicholas Seow Chiang Price

**Affiliations:** ^1^Department of Physiology and Neuroscience Program, Biomedicine Discovery Institute, Monash University, Clayton, Victoria, Australia; ^2^School of Engineering, RMIT University, Melbourne, Victoria, Australia

**Keywords:** eye movement, marmoset, motion, ocular following, sensory-motor transformation

## Abstract

Ocular following is a short-latency, reflexive eye movement that tracks wide-field visual motion. It has been studied extensively in humans and macaques and is an appealing behavior for studying sensory-motor transformations in the brain because of its rapidity and rigidity. We explored ocular following in the marmoset, an emerging model in neuroscience because their lissencephalic brain allows direct access to most cortical areas for imaging and electrophysiological recordings. In three experiments, we tested ocular following responses in three adult marmosets. First, we varied the delay between saccade end and stimulus motion onset, from 10 to 300 ms. As in other species, tracking had shorter onset latencies and higher eye speeds with shorter postsaccadic delays. Second, using sine-wave grating stimuli, we explored the dependence of eye speed on spatiotemporal frequency. The highest eye speed was evoked at ∼16 Hz and ∼0.16 cycles per degree (cpd); however, the highest gain was elicited at ∼1.6 Hz and ∼1.2 cpd. The highest eye speed for each spatial frequency was observed at a different temporal frequency, but this interdependence was not consistent with complete speed tuning of the ocular following response. Finally, we found the highest eye speeds when saccade and stimulus motion directions were identical, although latencies were unaffected by direction difference. Our results showed qualitatively similar ocular following in marmosets, humans, and macaques, despite over an order of magnitude variation in body and eye size across species. This characterization will help future studies examining the neural basis of sensory-motor transformations.

**NEW & NOTEWORTHY** Previous ocular following studies focused on humans and macaques. We examined the properties of ocular following responses in marmosets in three experiments, in which postsaccadic delay, spatial-temporal frequency of stimuli, and congruence of saccade and motion directions were manipulated. We have demonstrated short-latency ocular following in marmosets and discuss the similarities across three species that vary markedly in eye and head size. Our findings will help future studies examining the neural mechanism of sensory-motor transformations.

## Graphical abstract

**Figure:**
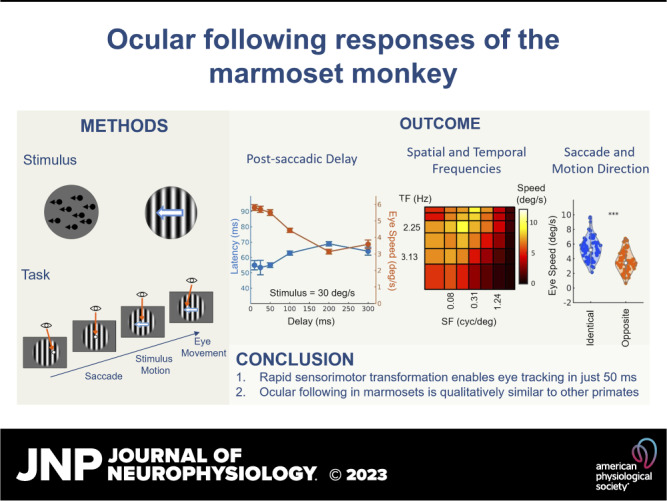

## INTRODUCTION

Animals need to continually process sensory information and generate motor responses to interact with the environment. This sensory-motor transformation is still not well understood, even though in some circumstances it is highly stereotyped and can reliably occur within tens of milliseconds. The ocular following response is a slow eye movement that almost immediately causes the eye to track the sudden movement of a large stimulus (1–3). It is useful for studying sensory-motor transformations because it is a stimulus-driven reflex that requires rapid sensory processing to extract visual motion information, which is then used to generate eye movements with a latency of just 60–80 ms. Whereas there has been extensive research on ocular following in macaques (1–6) and humans (7–12), it has not been examined in marmosets, a nonhuman primate animal model that has gained increasing interest in neuroscience research. The lissencephalic cerebral cortex of marmosets makes them an attractive model because most of its areas are accessible on the cerebral surface. In contrast, in macaques many areas, like the middle temporal cortex, are buried in the sulci and difficult to reach. Our aim was to examine ocular following in marmoset and characterize the stimulus properties that affect the latency and magnitude of the motor output. Parameterizing the behavioral aspects of ocular following will help constrain future studies using neural recordings to explore sensory-motor transformations in the brain.

Ocular following is proposed to be a compensatory reflex that helps stabilize the gaze on the visual scene after a moving observer looks off to one side (13). It is closely related to the early component of the optokinetic response (13, 14), a stabilization reflex evident in a wide range of species including those with less specialized cortical visual processing (e.g., rats and mice). Experimentally, a classical paradigm requires animals to first make a saccade from a peripheral position to a central target. After the saccade is completed, there is a delay period (i.e., postsaccadic delay) before the motion onset of a large-field visual stimulus in the background.

The eye tracking response is acutely stimulus dependent. The onset latency is negatively correlated with stimulus speed and contrast, whereas the eye speed is positively correlated with these two properties (3). When tested with sine-wave gratings with a range of spatial frequencies and temporal frequencies, the highest eye speed was evoked at ∼0.4 cycles per degree (cpd) and ∼20 Hz in macaques (15). In humans, the optimal spatial frequency for eye speeds varied across individuals, but the optimal temporal frequency was consistently 16 Hz between observers (8). In both humans and macaques, eye speeds have almost separable tunings for temporal and spatial frequencies of the stimuli, meaning that the preferred temporal frequency is consistent across different spatial frequencies (3, 8, 15). With increasing postsaccadic delay, initial eye speeds drop almost exponentially, whereas latency and later eye speeds (averaged eye speeds at 100–140 ms) are less affected (1, 8). The postsaccadic enhancement of eye speeds was suggested to be beneficial for suppressing postsaccadic ocular drifts (glissades).

Despite the short onset latencies, a multitude of cortical and subcortical areas are involved in generating a robust ocular following response. The pretectal nucleus of the optic tract (NOT) is a subcortical region that receives direct retinal projections and contains neurons predominantly tuned for temporo-nasal motion directions. Neurons in NOT fire before the ocular following response onset, their firing rates are well predicted by sensory inputs, and muscimol inactivation decreases eye velocity, suggesting a causal relationship between NOT activity and the ocular following response (16). Firing rates of individual neurons in the middle temporal area (MT) and the medial superior temporal area (MST) also ramp up before the ocular following responses start (17) and depend on stimulus speed (17) and postsaccadic delay (6, 18). MST neurons have higher sensitivities to motion stimuli at shorter postsaccadic delay, which was proposed to be causally related to lower eye speeds in behavioral response (18). Moreover, chemical lesions in MT and MST impair the magnitude of the eye movement response (19, 20), suggesting that these areas play a causal role in providing the sensory processing required for ocular following. It should be noted that the ocular tracking reflexes occur in species with less developed cortices, including rabbits (21) and mice (14), and in these species the role of the NOT is likely more important than that of MT/MST. Two subcortical regions, dorsolateral pontine nucleus (DLPN) and ventral paraflocculus (VPFL), also contribute processing critical to generating ocular following. DLPN is proposed to be an intermediate area that receives projections from MT/MST, further integrates sensory information, and projects to VPFL. VPFL is suggested to be responsible for generating motor commands (22). Understanding how stimulus parameters affect neural activities, and eventually affect oculomotor response, would aid in elucidating the neural mechanism for sensory-to-motor transformations. Given that the essential sensory area MT is buried in the sulcus in macaques, marmosets are particularly suited for studying the neural processing of ocular following.

It is unclear whether short-latency ocular following occurs in marmosets and, if it does, how the gains and latencies compare with those in macaques and humans. Marmosets are known to engage in head tracking (23), smooth pursuit eye movement (24), and eye tracking of optic flows (25). It is thus reasonable to expect they exhibit the ocular following response, which is also a slow eye tracking movement. However, their control of eye movement is poorer than that in macaques and humans, as reflected by lower gains and more frequent saccades in the smooth pursuit task (24). Therefore, it is important to characterize the differences between species.

In the present study, we tested three adult marmosets with a suite of stimuli that generate ocular following responses. We found reliable ocular following responses in all animals. Consistent with previous findings in humans and macaques, the latencies and speeds of ocular following responses in marmosets depend on stimulus properties. We found shorter latencies and higher eye speeds with shorter postsaccadic delays. The optimal spatiotemporal frequencies that evoked the highest eye speeds were similar to those found in humans and macaques, although the tuning for temporal frequency was more dependent on spatial frequency. We also explored the interaction of initial saccade direction and stimulus motion direction, which has not been systematically examined. It tells us how ocular following depends on the direction of the prior saccade, which is important for characterizing the functional significance of ocular following. We found higher eye speeds when the two directions were identical than when they were opposing. The results of this study will help future studies using advanced techniques such as multiarea electrophysiological recordings in the marmoset brain to disentangle the neural mechanisms underlying sensory-motor transformations (26).

## METHODS

### Animals

Three male adult common marmosets (*Callithrix jacchus*; *monkeys Bu, Br*, and *Ni*, 2, 2, and 6 yr old) participated in this study. *Monkey Ni* was part of a previous study involving saccades and fixations. All procedures were approved by the Monash Animal Research Platform Animal Ethics Committee and followed the Australian Code of Practice for the Care and Use of Animals for Scientific Purposes.

### Surgery

We implanted a titanium head post on each animal to stabilize its head during behavioral experiments. Surgery was performed under aseptic conditions. The animal was first injected with atropine (0.2 mg/kg im) and diazepam (2 mg/kg im). After 30 min, we induced anesthesia with alfaxalone (8 mg/kg im). The animal was placed in a stereotaxic frame and stabilized with earbars that had been covered in local anesthetic (2% xylocaine jelly). After intubation, the head was further stabilized with a palate bar and eyebars. Anesthesia was maintained with isoflurane (0.5–3%) in oxygen. Eyes were protected during surgery with liquid paraffin eye ointment. A midline incision was used to expose the skull, and up to six titanium screws (diameter 1.5 mm, length 4 mm) were inserted 1–1.5 mm into the skull. The exposed skull was coated with a thin layer of dental adhesive (Supabond, Parkell). A head post, which would stabilize the animal’s head during experiments, was then placed on the midline, and transparent dental acrylic (Ortho-Jet; Lang Dental Mfg. Co.) was applied to the base of the head post and the screws to secure them to the skull. The margin was sealed with surgical adhesive (VetBond; 3M). Animals received oral antibiotics for 7 days (cefalexin monohydrate, 30 mg/kg) and analgesia for 5 days (meloxicam, 0.2 mg/kg). *Monkeys Bu* and *Br* were also implanted with a titanium recording chamber in the same surgery, whereas *monkey Ni* had a separate surgery to implant a chronic multielectrode array in V1 of the right hemisphere.

### Stimulus and Procedure

Visual stimuli were generated in MATLAB (MathWorks, Natick, MA) with Neurostim (https://klabhub.github.io/neurostim/) and the Psychophysics Toolbox extension (27). Animals viewed the stimuli on a 24-in. Viewpixx/3D screen (refresh rate: 100 Hz; resolution: 1,920 × 1,080) with a fixed viewing distance of 56 cm.

**Figure 1.**
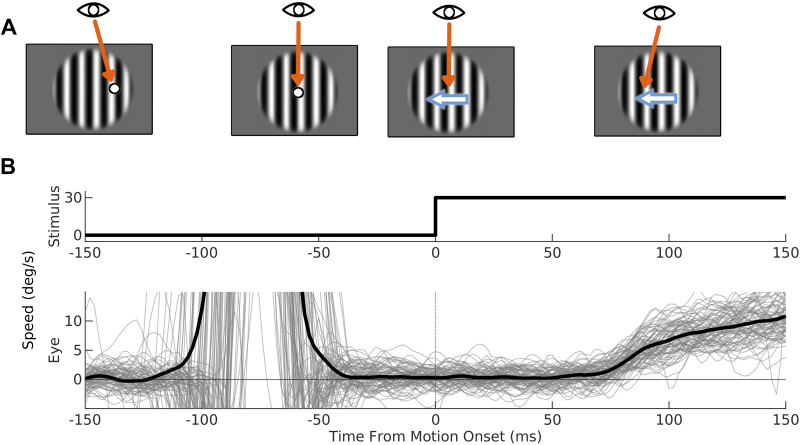
The ocular following paradigm. *A*: trials began with a large (28°) stationary stimulus (either a sinusoidal grating as shown or a random dot pattern) and a peripheral fixation target. Animals were required to fixate on the target for a randomized duration between 200 and 300 ms. Upon successful fixation, the target would disappear and reappear at the screen center. Animals were required to make a saccade to the new target within 500 ms. After the saccade, the stimulus would move after a postsaccadic delay (50 ms here). Eyes would track the stimulus motion soon after motion onset. *B*: stimulus and eye speed on 80 trials. For eye speed, the light gray lines represent data from individual trials and the black trace is the mean across trials.

We ran three experiments with identical procedures (Fig. 1). At the start of each trial, a stationary wide-field stimulus (a random dot pattern or sinusoidal grating with diameter 28°) appeared on the screen with a peripheral annulus target (diameter 1°). The peripheral target could appear 5° above, below, left, or right of the screen center. If the monkey’s eye stayed within 2° of the target for a random interval between 200 and 300 ms, the target would disappear and reappear at the screen center. Animals were required to make a saccade to within 2° of this central target within 500 ms. If they successfully completed the saccade, the central target would disappear and after a postsaccadic delay period of up to 300 ms the wide-field stimulus would move for 300 ms. Stimulus motion was followed by an intertrial interval of 1,000 ms during which a juice reward was delivered. If the centering saccade was not completed within 500 ms, the trial was terminated with no reward.

In *experiment 1*, we explored how postsaccadic delay affects tracking latency and speed. A random dot stimulus was used (2,000 black dots on a gray background, dot density 0.81 dots/°^2^, diameter 0.16°, dot luminance 0 cd/m^2^, background luminance 50 cd/m^2^, Michelson contrast 50%, 100% coherence, speed 30°/s). We randomly varied the postsaccadic delay with 6 levels (10, 30, 50, 100, 200, 300 ms). Saccade and stimulus directions were either leftward or rightward and always identical (e.g., the stimulus moved to the left following a leftward saccade).

In *experiment 2*, we examined how spatiotemporal frequency affects eye speeds and gains. Stimuli were 100% contrast sinusoidal gratings with six temporal frequencies (1.56, 3.13, 6.13, 12.25, 18.75, 25 Hz) and seven spatial frequencies (0.04, 0.08, 0.16, 0.31, 0.62, 1.24, 2.48 cpd). These values were identical to a previous study in macaque (15), allowing us to make a direct comparison between species. The spatial and temporal frequencies were randomized across trials. The circular edge of the grating was masked with a cosine function to avoid sharp contrast changes at the edges. The postsaccadic delay was fixed at 50 ms. As in *experiment 1*, saccade and stimulus directions were either leftward or rightward, and the two directions were always identical.

In *experiment 3*, we examined how the interaction between saccade and stimulus direction affects tracking latency and speed. The postsaccadic delay was fixed at 50 ms. We employed a random dot stimulus with the same parameters as in *experiment 1*. Four saccade directions (upward, downward, leftward, rightward) were tested. For each saccade condition, we had two relative stimulus directions that were either identical or opposite.

### Eye Velocity and Latency

Eye movements were tracked with a video-based EyeLink 1000 system (SR Research). Horizontal and vertical eye positions were recorded in both eyes at 500 Hz sampling rates, although only data from one eye were used for analysis. At the start of each session, eye positions were calibrated by presenting small marmoset faces at different locations to encourage fixation. A Savitzky–Golay filter with order 3 and length 42 ms (21 data points) was used to smooth the position data and calculate eye velocity.

To estimate the onset latency at which the eyes started moving, we aligned eye velocity with stimulus motion onset in each trial. Then we averaged the eye velocity across trials within the same experimental conditions at each time point. Because of the noisiness of data at the single-trial level, estimating latency in individual trials gave us unreliable results. Therefore, we fitted a two-part piecewise linear function (*Eq. 1*) to the trial-averaged eye speed traces at 30–150 ms after motion onset with the least-squares method.

(*1*) $$\begin{array}{c} y=ac+b\text{ if }t<c\\ y=at+b\text{ if }t\geq c \end{array}$$

The equation gives a constant speed before time *c* and a linear ramp of eye speed after *c*; parameter *c* defines the onset latency. We verified that determining latency with fixed acceleration thresholds (e.g., 40°g/s/s) gave us qualitatively similar results. Another method of latency estimation, which searches for the time when eye speed first deviates 1.5 SD above the mean speed at preresponse period, did not give us reliable measure of latency. This is probably due to the high level of noise in the eye speed signal in the preresponse period.

We employed a randomization test to determine the significance of the relationship between postsaccadic delay and onset latency. First, we shuffled the condition labels for all trials and estimated the latency from the trial-averaged traces for each pseudocondition. Then we found the slope of the simple linear regression model between latency and postsaccadic delay. By repeating this randomization procedure 1,000 times we obtained a null distribution of the slope.

### Two-Dimensional Gaussian Model

For each spatiotemporal frequency condition used in *experiment 2*, we computed the trial-averaged eye speed in three time windows of interest (60–110 ms, 80–130 ms, 100–150 ms). We then fit a two-dimensional (2-D) Gaussian function (*Eq. 2*) to the eye speeds, using the same approach as a previous study in macaque (15).

(*2*) $$y\left(\text{sf},\text{tf}\right)\;=\;A\cdot \text{exp}\left\{-{\left[\text{lo}g_{2}\left(\text{sf}\right)-\text{lo}g_{2}\left(sf_{0}\right)\right]}^{2}/\sigma_{s}^{2}\right\}\cdot \text{exp}\left[-{\left(\text{tf}-tf_{s}\right)}^{2}/\sigma_{t}^{2}\right]+b$$

where

$$tf_{s}=2^{Q\cdot \left[\text{lo}g_{2}\left(\text{sf}\right)-\text{lo}g_{2}\left(sf_{0}\right)\right]+\text{lo}g_{2}\left(tf_{0}\right)}$$

Parameters *A*, *b*, sf_0_, tf_0_, σ_s_, and σ_t_ are the gain, intercept, peak spatial frequency, peak temporal frequency, standard deviation of spatial frequency tuning, and standard deviation of temporal frequency tuning, respectively. Parameter *Q* quantifies the degree of dependence on spatial frequency. If *Q* = 1, the ocular following response is speed tuned; if *Q* = 0, the ocular following response is temporal frequency tuned. Therefore, the lower and upper bound of *Q* were set at 0 and 1, respectively. To determine the significance of *Q*, we performed an *F* test to compare the goodness of fit between a baseline model with *Q* = 0 fixed and the full model with *Q* as a free parameter. A significant *F* test result indicates that adding parameter *Q* improves the model fit.

## RESULTS

### *Experiment 1*: Onset Latencies and Eye Speeds Depend on Postsaccadic Delay

**Figure 2.**
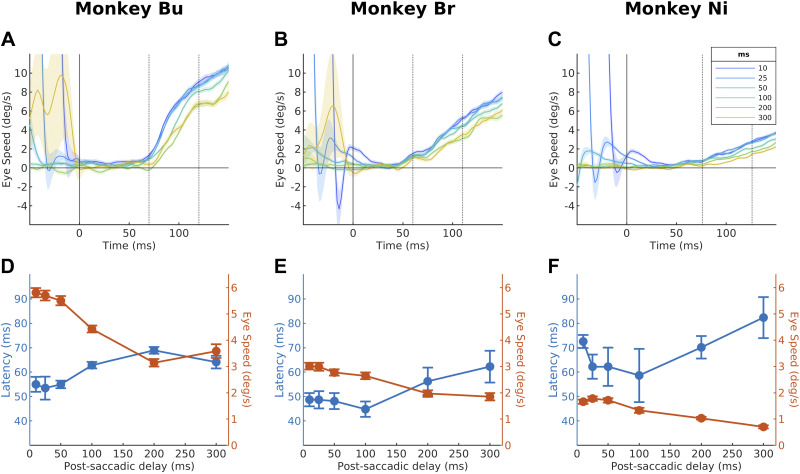
*A–C*: trial-averaged eye speeds over time for each animal. Each line shows the averaged eye speeds across trials for a single postsaccadic condition. Shaded region represents SE across trials. Dotted lines indicate animal-specific time windows for averaging eye speeds. *D–F*: latency (blue) and eye speed (orange) for each postsaccadic delay. Error bars indicate SD of the distribution of mean obtained with bootstrapping (*n* = 1,000). All conditions in all subjects had at least 30 trials. Mean (SD) of number of trials in each subject: *monkey Bu*: 67.3 (18.8); *monkey Br*: 70.7 (16.1); *monkey Ni*: 135.2(22.5).

In macaques and humans, the speed and onset latency of ocular following responses strongly depend upon postsaccadic delays; however, this has not been reported in other species. We first explored how postsaccadic delays affected the speed and latency of eye tracking in marmosets. At the start of each trial, a stationary, large-field random dot stimulus appeared in the background, with a small annulus target 5° left or right of the screen center overlaid. Animals were required to fixate on the peripheral target for a randomized period of 200–300 ms, after which the target would disappear and reappear in the screen center. Animals were then required to make a saccade to the new target within 500 ms. Upon completion of a saccade, the background stimulus moved for 300 ms after postsaccadic delays of 10–300 ms (Fig. 1). Eye speeds ramped up shortly after the motion onset (Fig. 2, *A*–*C*), although there was a large between-subject variation in gains. The maximum mean speeds were 6°/s, 3°/s, and 2°/s for *monkeys Bu*, *Br*, and *Ni*, respectively, corresponding to gains of 0.2, 0.1, and 0.03. We found consistent postsaccadic enhancement in all animals: onset latencies and eye speeds were positively and negatively correlated, respectively, with the postsaccadic delay (Fig. 2, *D*–*F*).

We found significant positive correlations between onset latencies and postsaccadic delays in all animals (Fig. 2*D*: *monkey Bu*: slope = 0.045 ms/ms, *P* < 0.001; Fig. 2*E*: *monkey Br*: slope = 0.050 ms/ms, *P* < 0.001; Fig. 2*F*: *monkey Ni*: slope = 0.052 ms/ms, *P* = 0.013). Onset latencies were determined by fitting a piecewise linear function (Eq. *1*) to the trial-averaged eye speed traces for each condition. We employed a randomization test to determine the significance of the relationship between postsaccadic delay and onset latency (see methods). Comparison of the empirical slope and null distribution showed significant positive slopes in all animals.

Eye speeds were negatively correlated with postsaccadic delays. Mean horizontal eye speed was calculated in 50-ms time windows starting 10 ms after the median onset latency across conditions for each animal (*monkey Bu*: 70–120 ms; *monkey Br*: 60–110 ms; *monkey Ni*: 76–126 ms). The 10-ms delay produces higher and more reliable estimates of single-trial speeds, but performing the analysis without this delay produces identical trends. We then conducted a randomization test as described for latencies, which showed a significant slope in the relationship between postsaccadic latency and eye speed in all animals (*monkey Bu*: slope = −9.13°/s^2^, *P* < 0.001; *monkey Br*: slope = −4.34°/s^2^, *P* < 0.001*; monkey Ni*: slope = −3.72°/s^2^, *P* < 0.001). A limitation of calculating eye speed in a fixed time window is that the eye is moving slower at the start of the averaging window when the postsaccadic delay is longer. To address this, we also determined that the relationship between postsaccadic delay and eye speed had significant regression and slope when eye speed was calculated in a 50-ms window defined relative to the mean latency for each postsaccadic delay condition (*monkey Bu*: slope = −2.89°/s^2^, *P* < 0.001; *monkey Br*: slope = −1.65°/s^2^, *P* = 0.0284; *monkey Ni*: slope = −2.46°/s^2^, *P* < 0.001). To conclude, higher eye speeds and shorter latencies were found with shorter postsaccadic delays, which is consistent with findings in humans and macaques.

### *Experiment 2*: Eye Speeds and Gains Depend on Spatiotemporal Frequencies

**Figure 3.**
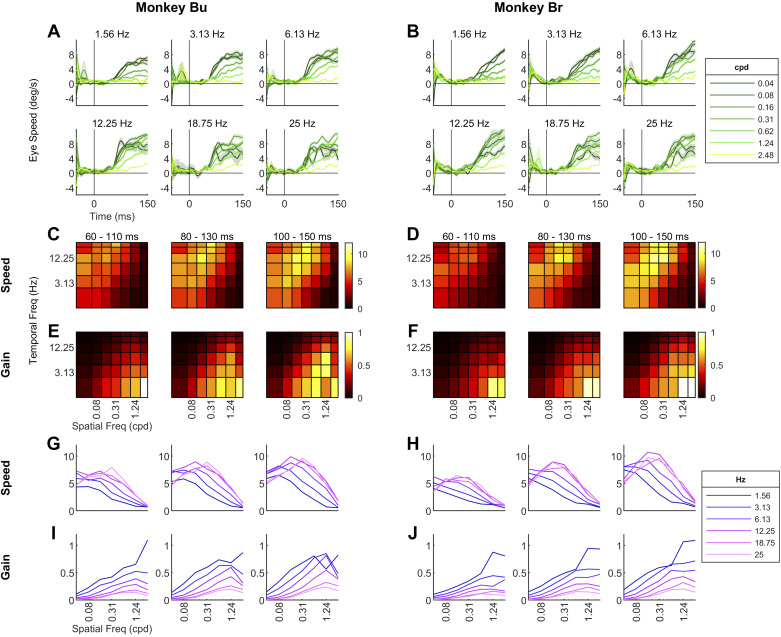
*A* and *B*: trial-averaged eye speeds over time for 2 animals separately plotted for each temporal frequency condition. Each line represents a different spatial frequency (cycles per degree, cpd). Darker color indicates lower spatial frequency. *C* and *D*: heat maps of eye speeds at three 50-ms time windows (60–110 ms, 80–130 ms, 100–150 ms). Each cell represents a spatiotemporal condition, and the color indicates eye speed; *x*- and *y*-axes are in log scale. *E* and *F*: heat maps of gains at three 50-ms time windows. Gain is defined as eye speed divided by stimulus speed. *G* and *H*: eye speeds at 3 time windows with spatial frequency as the horizontal axis. Each line represents a different temporal frequency. *I* and *J*: gains plotted with spatial frequency as the horizontal axis. All conditions in all subjects had at least 14 trials. Mean (SD) of number of trials in each subject: *monkey Bu*: 36.8 (7.4); *monkey Br*: 31.3 (7.5). Data from *monkey Ni* are not included because of failure to trigger ocular following in 16/42 conditions.

Previous studies in humans and macaques found a strong dependence of eye speed on spatiotemporal frequency. Therefore, we examined how the spatial and temporal frequency of a grating stimulus influenced eye speed and gain. The procedure was identical to *experiment 1* except that we presented sine-wave gratings instead of random dot stimuli. We selected seven spatial (0.04–2.48 cpd) and six temporal (1.56–25 Hz) frequencies, giving us 42 stimuli in total. *Monkey Ni* had lower gains than the other subjects in *experiment 1*. Therefore, it may not be surprising that we could not observe reliable ocular following responses in 16/42 stimuli. With <20 valid trials in those conditions, it was hard to estimate eye speeds reliably. Therefore, *monkey Ni*’s results are not reported here. In *monkeys Bu* and *Br*, the optimal spatiotemporal frequencies that elicit the highest eye speeds and gains were consistent across time (Fig. 3).

Since it was unclear how the spatiotemporal frequency tuning or the optimal frequency shifts over time, we examined eye speeds in three time windows (60–110 ms, 80–130 ms, 100–150 ms). The optimal spatial and temporal frequencies were consistently ∼0.16 cpd and ∼16 Hz across these time windows in both animals (Fig. 3, *C* and *D*). To examine whether eye speeds were tuned to speed or separately to spatial and temporal frequency, we fit a 2-D Gaussian model to each time window in each animal and examined the parameter that quantifies the degree of dependence on spatial frequency (*Q* in *Eq. 2*). A value of *Q* = 0 means that the spatial and temporal frequency tuning are separable, and a value of *Q* = 1 indicates that the data are tuned to speed independent of spatial frequency. The estimated values of *Q*, in the order of early to late time windows, were 0.263, 0.246, and 0.215 in *monkey Bu* and 0.225, 0.225, and 0.268 in *monkey Br*, respectively. The model had good fits in all time windows in both animals (*R*^2^ > 0.890). Although these values were closer to 0 than 1 numerically, it is necessary to test whether these *Q* values are significantly different from 0. We used an *F* test to determine whether the full model, with *Q* as a free parameter, fit the data better than a reduced model with fixed *Q* = 0. All *Q* values were significantly different from 0 under the *F* test (*P* < 0.001), suggesting interdependence between tunings of spatial and temporal frequency.

Gain is defined as the ratio of eye speed and stimulus speed. The optimal spatial and temporal frequencies associated with the highest gains were consistent across time in both animals at ∼1.24 cpd and ∼1.56 Hz (Fig. 3, *E* and *F*). We did not fit the 2-D Gaussian model to gains because the estimate of *Q* is hard to interpret when the optimal stimulus is close to the edge of the range of tested conditions. Gains were highest with low stimulus speeds (high spatial frequencies and low temporal frequencies), suggesting that it is challenging to choose a stimulus that produces oculomotor responses with both high gain and high absolute speed.

### *Experiment 3*: Eye Speeds, but Not Latencies, Depend on the Congruence of Saccade and Stimulus Direction

**Figure 4.**
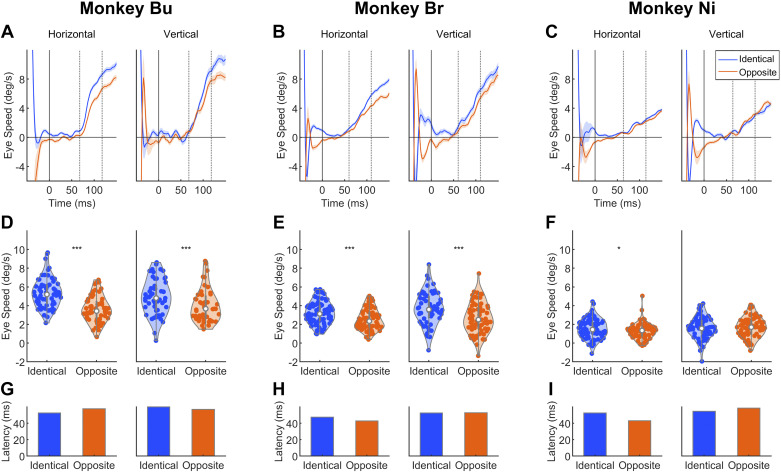
*A–C*: trial-averaged eye speeds for each animal for identical (blue) and opposite (orange) conditions, shown separately for horizontal and vertical components. Dotted lines indicate time windows used to calculate eye speeds. *D–F*: comparison of eye speeds in identical and opposite conditions. Each dot represents the mean eye speed in a single trial. Significance level tested with *t* tests: ***P < 0.001; *P < 0.05. *G–I*: comparison of latencies in identical and opposite conditions. A randomization test with condition label shuffling was conducted to test significance. No conditions were associated with significant differences in latency. All conditions in all subjects had at least 47 trials. Mean (SD) of number of trials in each subject: *monkey Bu*: 31.9 (6.2); *monkey Br*: 36.6 (4.7); *monkey Ni*: 38.0 (2.4).

Although the ocular following paradigm commonly involves a saccade preceding stimulus motion onset, it is not clear how the saccade and stimulus directions interact. Examining this interaction is important for understanding how ocular following depends on the prior saccade. We tested ocular following with four saccade directions (up, down, left, right), each with two motion directions that were identical or opposite to the saccade direction. For example, a leftward saccade direction could be paired with leftward (180°; identical) or rightward (0°; opposite) stimulus motion. We averaged the results from conditions with saccade directions in the horizontal and vertical axes after inverting the eye speeds in conditions with stimulus directions of 180° and 270° (Fig. 4, *A*–*C*). Eye speeds were higher when saccade and stimulus directions were identical than when they were opposite for both vertical and horizontal components. However, direction similarity had no effect on latencies.

Eye speeds generally depended on direction similarity. For *monkey Bu*, mean eye speeds were significantly higher in identical than in opposite conditions in both horizontal [Fig. 4*D*; *t*(62) = 6.08, *P* < 0.0001] and vertical [Fig. 4*D*; *t*(46) = 3.90, *P* < 0.001] components. A similar enhancement was observed in *monkey Br* [Fig. 4*E*; horizontal: *t*(72) = 3.80, *P* < 0.001; vertical: *t*(62) = 3.92, *P* < 0.001]. For *monkey Ni*, the effect was significant in the horizontal component [Fig. 4*F*; *t*(74) = 2.04, *P* = 0.045) but not in the vertical component [Fig. 4*F*; *t*(72) = −0.698, *P* = 0.487]. As in *experiment 1*, eye speeds were calculated as the mean amplitude in animal-specific 50-ms time windows (*monkey Bu*: 68–118 ms; *monkey Br*: 60–110 ms; *monkey Ni*: 64–114 ms).

There were no significant differences in latencies between identical and opposite conditions in both horizontal (Fig. 4*G*: *monkey Bu*: 5 ms, *P* = 0.142; Fig. 4*H*: *monkey Br*: −5 ms, *P* = 0.136; Fig. 4*I*: *monkey Ni*: −10 ms, *P* = 0.226) and vertical (Fig. 4*G*: *monkey Bu*: –3 ms, *P* = 0.174; Fig. 4*H*: *monkey Br*: 0 ms, *P* = 0.471; Fig. 4*I*: *monkey Ni*: 4 ms, *P* = 0.348) components. As in *experiment 1*, we employed a randomization test to test significance. We shuffled the condition labels 1,000 times, estimated latency of each pseudocondition, and calculated the latency difference between conditions. This formed a null distribution of latency difference, which we compared with the empirical values to assess significance.

## DISCUSSION

In this study, we found three main results. First, postsaccadic enhancement of the ocular following response was evident in marmosets, reflected by shorter latencies and higher eye speeds with shorter postsaccadic delays. Second, eye speeds and gains were dependent on the spatiotemporal frequencies of the stimuli. Finally, identical saccade and stimulus directions evoked higher eye speeds than the opposite conditions, whereas direction difference had no significant effect on latencies.

Our findings in *experiment 1* were qualitatively similar to previous reports in macaques and humans. First, the ocular following responses have ultrashort latencies, although the latencies are even shorter in marmosets (∼50 ms) than in macaques (∼60 ms) and humans (∼80 ms). This is not surprising given that marmosets have considerably smaller eyes and brains, with the corresponding shorter axons suggesting that faster responses are possible. Second, our three monkeys show markedly different gains (20%, 10%, and 3%) for the random dot patterns with identical parameters. These values are comparable to those found in macaques (∼10–30%) and humans (∼5–14%). Likewise, previous macaque and human studies (3, 8) have also reported large interindividual variations in gains for ocular following responses. Human studies of smooth pursuit eye movements have also found dramatic differences in gains across individuals (28), although some of these differences may reflect practice effects (29, 30). Finally, the dependence of eye speed on postsaccadic delay suggests that the ocular following response in marmosets may have a functional role similar to that hypothesized in humans and macaques. Eyes do not always stop at a designated target at the end of saccade and may instead drift forward or backward by a fraction of a degree. This ocular drift, which is called a glissade, reduces the stability of the visual scene on the retina and reduces spatial acuity. By enhancing the gain of ocular following and shortening the latency until it begins in the period shortly after saccades, the visual system can counteract glissades (1, 6).

The spatiotemporal frequencies that evoked the highest eye speeds in marmosets were similar to those previously reported in macaques and humans. In our study, gratings of ∼0.16 cpd and ∼16 Hz were optimal, compared with ∼0.4 cpd and ∼20 Hz in macaques (15). We note that the high-speed stimuli in *experiment 2* failed to trigger ocular following in one monkey, so these data are based on two subjects. The lower optimal spatial frequency in marmosets than in macaques is consistent with previous comparison of tunings in MT cells in the two primates (31). The optimal spatial frequency in humans varied a lot across individuals, and thus no reliable estimates have been reported, whereas the optimal temporal frequency is consistently ∼16 Hz across observers (8). It is not clear why the optimal temporal frequencies are similar across species. However, it should be noted that because of the 100 Hz refresh rate of our monitor we did not present gratings with temporal frequencies higher than 25 Hz. Therefore, there is a possibility that the optimal temporal frequency in marmosets actually sits outside the range we tested. On a related note, our range of spatial frequencies is not ideal since we could not see an obvious falloff of eye speeds in the low-spatial frequency conditions. Using stimuli with lower spatial frequencies may give us a more complete picture.

Our findings suggest that the ocular following responses in marmosets are more speed tuned than those in macaques and humans. Tunings of temporal and spatial frequency are mostly separable in macaques, as reflected by an estimated value of 0 for the parameter *Q* in the 2-D Gaussian model (15). There are no human data reported with 2-D Gaussian fitting. However, we could fit the model with the results reported from an experiment testing the ocular following response with gratings of five spatial frequencies and five temporal frequencies (8). Using the data from two subjects, we obtained *Q* values of 0.0261 and 0.144, which were not significantly different from 0 under the *F* test. Our estimated *Q* values (0.215–0.268) were higher, meaning that our results contained more speed tuning in responses than has been reported for macaques and humans. Moreover, the 2-D Gaussian model with *Q* as a free parameter had significantly better fits than the baseline model with *Q* value fixed at 0, which suggests that tunings of temporal and spatial frequencies are not completely separable. Previous studies in the tuning of MT cells in macaques revealed that many neurons had at least intermediate tuning for temporal and spatial frequency (32, 33). However, most MT cells in marmosets showed separable selectivity for temporal and spatial frequency (31), which is contradictory to our findings in the oculomotor response. Future electrophysiological studies will be needed to clarify the relationship between stimulus representations in MT and ocular following behavior.

The enhancement of eye speeds with identical saccade and stimulus directions is consistent with findings on the effect of saccadelike stimulus, which is a high-speed stimulus that mimics the retinal input during saccade. By introducing a saccadelike stimulus before a test stimulus that actually triggers ocular following response, it was found that eye speeds were higher when the saccadelike stimulus moved in the opposite direction of the test stimulus (1). Since a real saccade with the identical direction as the stimulus creates retinal input similar to saccadelike stimulus in the opposite direction, our findings were consistent with this study. A possible explanation for this phenomenon is the involvement of attentive pursuit (34). When the saccade is in the same direction as stimulus motion, it may be assumed that the saccade serves to foveate a particular feature, which triggers the attentive pursuit system (35). In other words, smooth pursuit may be involved in the identical directions condition, whereas the opposite directions condition remains unaffected by pursuit. However, in our paradigm the saccade occurs over a stationary stimulus, which means attention can only take effect within a short time window after saccade onset and before it ends.

A limitation of this study is that we did not test the full range of stimulus properties that have been examined in the ocular following studies in macaques. Instead, we replicated the major features of ocular following, including postsaccadic enhancement and dependence on spatiotemporal frequencies. The other properties of ocular following, for example dependence on eccentricity (3, 8), binocular disparity (12, 36, 37), and pattern motion (5, 9, 10), should also be investigated in marmosets.

Our findings will help future studies in examining the neural mechanism of ocular following. Comparative analyses showed that the basic response properties of MT cells are similar in marmosets and macaques (38). Areas MT and MST are buried within the superior temporal sulcus in macaques. In marmosets, these areas are fully accessible on the cortical surface. Using marmosets as an alternative animal model therefore opens opportunities for applying contemporary technologies, including extracellular recording with multielectrode arrays, two-photon calcium imaging of population activities, and precise inactivation of cells with optogenetics, etc. Furthermore, our results provide a quantitative basis for parameter selection. If maximizing eye speed is important, we recommend using stimuli with spatial frequency ∼0.16 cpd, temporal frequency ∼16 Hz, and postsaccadic delays < 50 ms.

In summary, our results showed that ocular following responses in marmosets depend on postsaccadic delay, spatial and temporal frequencies of the stimulus, and the similarity between saccade and stimulus direction. These results can be used to design experiments that investigate sensory-to-motor mechanisms in the marmoset monkey.

## DATA AVAILABILITY

Data and analysis scripts can be found on https://doi.org/10.5281/zenodo.8077848.

## GRANTS

This project was funded by the Australian Research Council (DP2101002107 to N.S.C.P.; DE180100344 to M.A.H.; DP210103865 to S.L.C) and by the National Health and Medical Research Council of Australia (APP1185442 to M.A.H.). H.M.K.Y. was funded by Monash Graduate Scholarship.

## DISCLOSURES

No conflicts of interest, financial or otherwise, are declared by the authors.

## AUTHOR CONTRIBUTIONS

H.M.K.Y. and N.S.C.P. conceived and designed research; H.M.K.Y., T.J.A.-W., S.L.C., M.A.H., and N.S.C.P. performed experiments; H.M.K.Y. analyzed data; H.M.K.Y. and N.S.C.P. interpreted results of experiments; H.M.K.Y. prepared figures; H.M.K.Y. and N.S.C.P. drafted manuscript; H.M.K.Y., T.J.A.-W., S.L.C., M.A.H., and N.S.C.P. edited and revised manuscript; H.M.K.Y., T.J.A.-W., S.L.C., M.A.H., and N.S.C.P. approved final version of manuscript.
